# An IBD-based mixed model approach for QTL mapping in multiparental populations

**DOI:** 10.1007/s00122-021-03919-7

**Published:** 2021-08-03

**Authors:** Wenhao Li, Martin P. Boer, Chaozhi Zheng, Ronny V. L. Joosen, Fred A. van Eeuwijk

**Affiliations:** 1 Biometris, Wageningen University and Research Center, P.O Box 100, 6700 AC Wageningen, The Netherlands; 2 Rijk Zwaan Breeding B.V., P.O Box 40, 2678 ZG De Lier, The Netherlands

## Abstract

**Key message:**

The identity-by-descent (IBD)-based mixed model approach introduced in this study can detect quantitative trait loci (QTLs) referring to the parental origin and simultaneously account for multilevel relatedness of individuals within and across families. This unified approach is proved to be a powerful approach for all kinds of multiparental population (MPP) designs.

**Abstract:**

Multiparental populations (MPPs) have become popular for quantitative trait loci (QTL) detection. Tools for QTL mapping in MPPs are mostly developed for specific MPPs and do not generalize well to other MPPs. We present an IBD-based mixed model approach for QTL mapping in all kinds of MPP designs, e.g., diallel, Nested Association Mapping (NAM), and Multiparental Advanced Generation Intercross (MAGIC) designs. The first step is to compute identity-by-descent (IBD) probabilities using a general Hidden Markov model framework, called reconstructing ancestry blocks bit by bit (RABBIT). Next, functions of IBD information are used as design matrices, or genetic predictors, in a mixed model approach to estimate variance components for multiallelic genetic effects associated with parents. Family-specific residual genetic effects are added, and a polygenic effect is structured by kinship relations between individuals. Case studies of simulated diallel, NAM, and MAGIC designs proved that the advanced IBD-based multi-QTL mixed model approach incorporating both kinship relations and family-specific residual variances (*IBD.MQMkin_F*) is robust across a variety of MPP designs and allele segregation patterns in comparison to a widely used benchmark association mapping method, and in most cases, outperformed or behaved at least as well as other tools developed for specific MPP designs in terms of mapping power and resolution. Successful analyses of real data cases confirmed the wide applicability of our IBD-based mixed model methodology.

**Supplementary Information:**

The online version contains supplementary material available at 10.1007/s00122-021-03919-7.

## Introduction

MPP designs have their unique advantages for QTL mapping over biparental populations and association panels. Crossing two parents in a biparental population can balance allele frequencies and increase the chance to detect rare QTLs, but the narrow genetic diversity from only two parents limits the number of detected QTLs (Liu and Zeng 2000; Pascual et al. 2016). We can use association panels to broaden the genetic diversity, but low-frequency variants may increase false positives (Malosetti et al. 2007; Xiao et al. 2016), and the potential population structure may mask the effects of causal variants (Flint-Garcia et al. 2003; Malosetti et al. 2007; Xiao et al. 2016; Sul et al. 2018). Experimental MPP designs, as a compromise between biparental populations and association panels, show broad genetic diversity with a controlled population structure. Such MPP designs like diallel (Giraud et al. 2017; Turner et al. 2018), NAM (Yu et al. 2008), and MAGIC (Huang et al. 2011, 2015; Gardner et al. 2016) populations have been proved to be promising populations for QTL mapping.

QTL mapping models can be classified into family-based (or linkage) and population-based (or linkage disequilibrium) approaches based on the specific design (Myles et al. 2009; Würschum 2012; Xu et al. 2017). Most studies comparing and evaluating different statistical models were restricted to only one specific MPP design, such as a sugar beet random cross design (Würschum et al. 2011, 2012), a maize NAM population (Li et al. 2011; Giraud et al. 2014), and a tomato MAGIC population (Pascual et al. 2015). Several requirements can be formulated for the QTL mapping methodology involving general MPP designs. Firstly, it will be convenient to define QTL effects in terms of their origins while allowing residual polygenic and non-genetic effects to have heterogeneous variances. The multi-QTL effect (MQE) model with a mixture of bi-allelic, ancestral, and parental QTL effects and cross-specific residual proposed by Garin et al. (2017) provides an example of such an approach for the EU-NAM maize data collection. Secondly, in addition to a kinship or marker structured polygenic term to control background genetic variation, it is attractive to have further control using some form of cofactors as in classical composite interval mapping. A good example of the latter is the inclusive composite interval mapping (ICIM) approach as described by Li et al. (2007), Zhang et al. (2019), and Shi et al. (2019), with an application to an eight-way MAGIC design in cow pea.

In the current paper, we aim at developing a unified QTL mapping framework compatible with all kinds of MPPs, including diallel, NAM, and MAGIC designs. Our proposal combines strategies from family-based and population-based mapping approaches. Family-based QTL mapping approaches were developed for biparental populations to detect bi-allelic QTLs. In the context of MPP designs, we can estimate multiallelic effects referring to different parental origins. Despite differences in MPP designs, parental origins of offspring alleles can always be estimated as functions from IBD probabilities between parents and offspring. To infer the precise genome composition inherited from parents to progenies, we need a sophisticated approach for IBD computation using the pedigree and whole-genome information. As IBD computations for specific MPP designs can be tedious and error-prone, a general pipeline is required (Broman et al. 2018). Our study applied a general approach called RABBIT for IBD computations supporting QTL mapping for any MPP design (Zheng et al. 2015, 2018). The IBD information forms the basis for creating design matrices, or genetic predictors, to which QTL allele effects can be estimated. Additional terms are added to model random genetic effects from families, like in (Garin et al. 2017), and a polygenic effect that is structured by kinship similar to what is commonly done in genome-wide association studies (GWAS) (Stich et al. 2008; Malosetti et al. 2011).

We constructed a series of models varying in whether they adopt identity-by-state (IBS) or IBD information as the basis for genetic predictors and how they account for individual relatedness. We simulated diallel, NAM, and MAGIC designs from four inbred Arabidopsis parents to evaluate the performance of our models and compared that performance where possible with that of alternative approaches, such as IciMapping (Meng et al. 2015) for our simulated NAM design, and the integrated genetic analysis software for multiparent pure-line population (GAPL) (Zhang et al. 2019) for the simulated MAGIC design. We compared further QTL mapping results obtained by our IBD-based mixed model approach for our simulated diallel and NAM designs with those of mppR (Garin et al. 2017). Overall, for the simulated data our IBD-based mixed model approach performed well for mapping power and resolution and was competitive in comparison to alternative approaches, where our method is applicable to a wider set of MPPs. Further demonstrations of our approach are provided for various empirical MPP design datasets.

## Methodology

We developed the QTL mapping methodology in the linear mixed model framework. First, a linkage map for the MPP design is required as input for IBD calculations and QTL identification procedure. Best is to construct a consensus map following a protocol of marker cleaning, grouping, ordering (Wu et al. 2008; Taylor 2018) and map integration (Endelman and Plomion 2014) for MPP designs. An MPP design contains $N$ individuals in $F$ families that are derived from crosses between $P$ parents. The contribution of a putative QTL to the phenotype is given by the product of an $N\times P$ design matrix $M$ and a $P\times 1$ vector $a$ of genetic effects. The element ${M}_{ij}$ represents the genetic predictor for the change of the phenotype in the ${i{th}}$ *(i* = *1, 2, …, N)* individual contributed by an offspring allele stemming from the ${j{th}}$ *(j* = *1, 2, …, P)* parent. We constructed two types of models, depending on how the genetic predictor was calculated. Specifically, in the IBS-based model, the genetic predictors are given by observed numbers of IBS alleles. In the IBD-based models, the genetic predictors are given by the expected numbers of IBD alleles, where the QTL allelic effect, ${a}_{j}$, will denote the haplotype effect of the ${j{th}}$ parent.

### IBD probability calculation

**Fig. 1:**
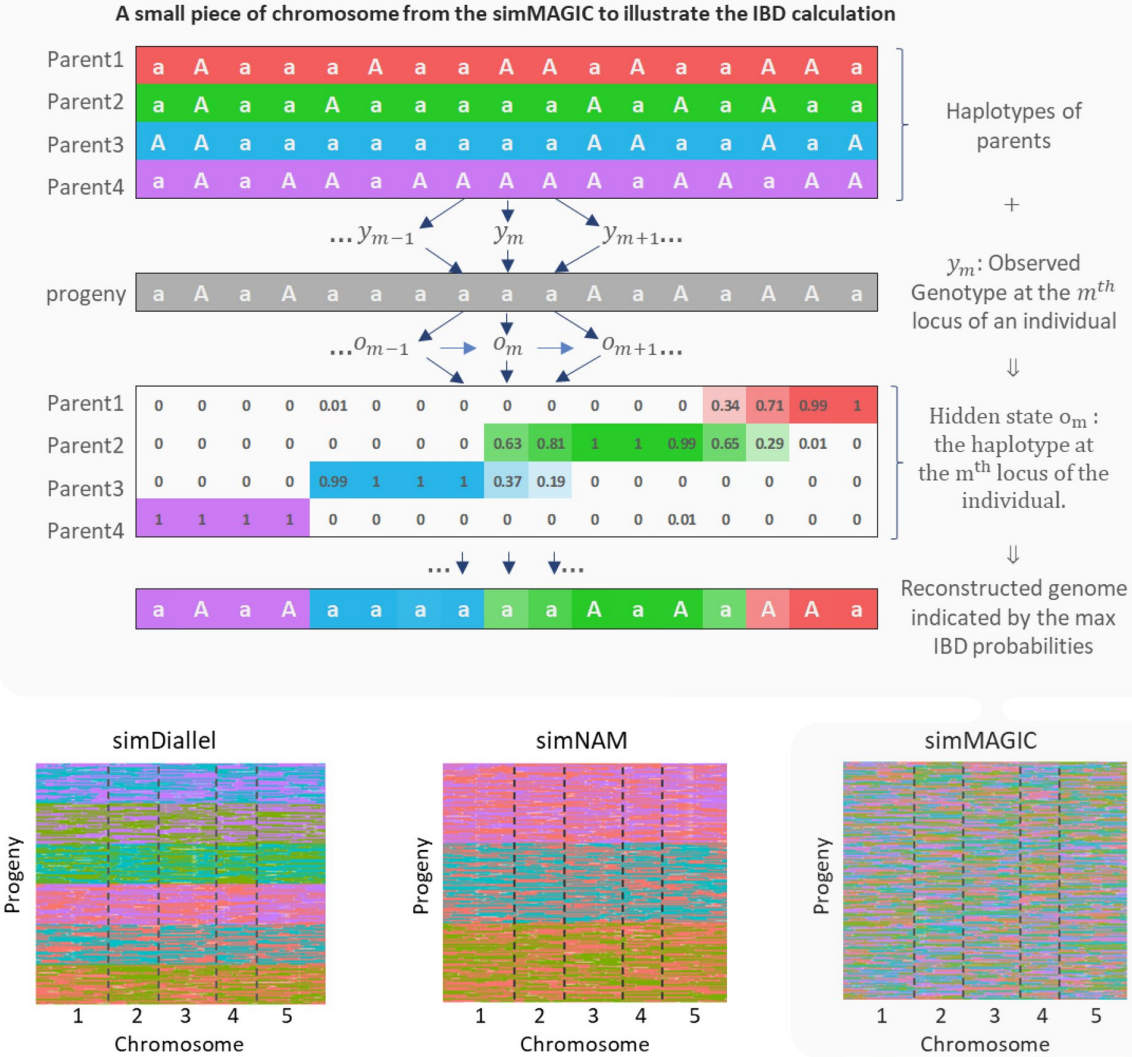
**Upper panel** An example for a MAGIC design to illustrate the framework of IBD calculations underlying the construction of design matrices for mixed model QTL mapping. The assessment of chromosome segments in the offspring of having been transmitted from one of the parents follows on the estimation of transmission probabilities of alleles from parents to offspring. For this example, MAGIC parents and offspring are assumed to be inbred. Therefore, haplotypes and genotypes coincide with respect to allelic composition. The labels 1 and 2 refer to alternative alleles in the parental haplotypes and to alternative genotypes in the offspring.  For the hidden parental states in the offspring, the transmission probabilities for respective parents are shown. The parental contribution with highest transmission probability determines the IBD status in the offspring individuals. **Lower panel** Graphical genotype heat maps showing parent of origin information for offspring in simulated diallel, NAM, and MAGIC populations obtained from thresholding IBD probabilities

The genetic predictors in the IBD-based models were calculated using the RABBIT software (Zheng et al. 2015). RABBIT calculates the genetic predictors by haplotype reconstruction within the hidden Markov model (HMM) framework, where the prior transition probability matrix for modeling how the hidden states change along chromosomes can be calculated using a recursive algorithm on the breeding pedigree (Zheng et al. 2018). The flexibility of RABBIT follows from the applicability of this recursive algorithm to arbitrarily fixed pedigrees, and the genotypic data model can account for genotyping errors and missing values in parents and offspring. The principal outputs of RABBIT are the posterior probabilities of the hidden IBD states for each offspring at each locus, conditional on the genotypic data at all loci. For homozygous populations with inbred parents, the hidden states are the parental origins, and the genetic predictors are given by twice the parental origin probabilities. For heterozygous populations with inbred parents, the hidden states are given by the pairwise combinations of the parental origins, and the exported posterior probabilities can be easily transformed into the parental origin probabilities. Figure 1 gives a schematic impression of the IBD calculations performed by RABBIT.

### Mixed model construction

We constructed five IBD-based models (Table 1) to estimate multiallelic effects in a genome-wide scan. We compared the IBD-based modeling with a benchmark GWAS model based on IBS information. The models are defined for phenotypic data vectors coming in as genotypic means, best linear unbiased estimates, or BLUEs, obtained from a preliminary phenotypic analysis of trial data accounting for experimental design factors and spatial trends. We expected that for all models that incorporated cofactor and kinship corrections, the residual term $\varepsilon$ would principally represent non-genetic within-trial variation.

**Table 1:** Overview of mixed models used for IBD-based QTL mapping

Model name	Genetic predictor	Genome background	Residual structure	Formula	VCOV structure of random terms
IBD.SQM_U	IBD	–	Homogeneous (Uniform)	$$Y = X\beta + M_{q} a_{q} + \varepsilon$$	$$a_{q} \sim N\left( {0,I_{P} \sigma_{q}^{2} { }} \right)$$$$\varepsilon \sim N\left( {0,{ }I_{N} \sigma_{\varepsilon }^{2} } \right)$$
IBD.SQM_F	IBD	–	Family-specific	$$Y = X\beta + M_{q} a_{q} + \varepsilon$$	$$a_{q} \sim N\left( {0,I_{P} \sigma_{q}^{2} { }} \right)$$$$\varepsilon \sim { }N\left( {0, \oplus_{k = 1}^{F} I_{{n_{k} }} \sigma_{{\varepsilon_{k} }}^{2} } \right)$$
IBD.MQM_F	IBD	Cofactors	Family-specific	$$Y = X\beta + \mathop \sum \limits_{c \ne q} M_{c} a_{c} + M_{q} a_{q} + \varepsilon$$	$$a_{q} \sim N\left( {0,I_{P} \sigma_{q}^{2} { }} \right)$$$$a_{c} \sim N\left( {0,I_{P} \sigma_{c}^{2} { }} \right)$$$$\varepsilon \sim { }N\left( {0, \oplus_{k = 1}^{F} I_{{n_{k} }} \sigma_{{\varepsilon_{k} }}^{2} } \right)$$
IBD.Kin_F	IBD	Polygenic term	Family-specific	$$Y = X\beta + M_{q} a_{q} + g + \varepsilon$$	$$a_{q} \sim { }N\left( {0,I_{P} \sigma_{q}^{2} } \right)$$$$g\sim { }N\left( {0,K\sigma_{g}^{2} } \right)$$$$\varepsilon \sim { }N\left( {0, \oplus_{k = 1}^{F} I_{{n_{k} }} \sigma_{{\varepsilon_{k} }}^{2} } \right)$$
IBD.MQMkin_F	IBD	Cofactors Polygenic term	Family-specific	$$Y = X\beta + \mathop \sum \limits_{c \ne q} M_{c} a_{c} + M_{q} a_{q} + g + \varepsilon$$	$$a_{q} \sim N\left( {0,I_{P} \sigma_{q}^{2} { }} \right)$$$$a_{c} \sim N\left( {0,I_{P} \sigma_{c}^{2} { }} \right)$$$$g\sim { }N\left( {0,K\sigma_{g}^{2} } \right)$$$$\varepsilon \sim { }N\left( {0, \oplus_{k = 1}^{F} I_{{n_{k} }} \sigma_{{\varepsilon_{k} }}^{2} } \right)$$
IBS.Kin (GWAS model)	IBS	Polygenic term	Homogeneous (Uniform)	$$Y = X\beta + X_{q} \lambda_{q} + g + \varepsilon$$	$$g\sim { }N\left( {0,K\sigma_{g}^{2} } \right)$$$$\varepsilon \sim N\left( {0,{ }I_{N} \sigma_{\varepsilon }^{2} } \right)$$

The first two models are called *IBD.SQM_U* and *IBD.SQM_F*. We use IBD information as the basis for the genetic predictors in a simple single-locus QTL mapping model (SQM) with, respectively, homogeneous, or uniform (_U), and family-specific (_F) variance–covariance (VCOV) structures on residual terms. The first model *IBD.SQM_U* can be expressed as:

$$Y = X\beta + M_{q} a_{q} + \varepsilon$$

$$a_{q} \sim N\left( {0,I_{P} \sigma_{q}^{2} } \right)$$

$$\varepsilon \sim N\left( {0, I_{N} \sigma_{\varepsilon }^{2} } \right),$$

where $Y$ is the $N\times 1$ column vector for the phenotypes of $N$ individuals; $X$ is the $N\times F$ design matrix with elements 1 or 0 indicating whether the ${i{th}} (i=\mathrm{1,2},\dots ,N)$ individual belongs to the ${k{th}} (k=\mathrm{1,2},\dots ,F)$ family or not, and $\beta$ is the $F\times 1$ column vector of fixed family intercept effects; ${M}_{q}$ is the $N\times P$ design matrix containing the expected number of parental alleles obtained by taking two times the IBD probability between parent and offspring at a putative QTL position, indexed by the subscript *q*. The $P\times 1$ column vector ${a}_{q}$ contains the random parental effects at the putative QTL with VCOV structure equal to ${I}_{P}{\sigma }_{q}^{2}$, ${\sigma }_{q}^{2}$ being the genetic variance of the QTL effect; $\varepsilon$ is the residual term with a homogeneous VCOV structure expressed as ${I}_{N}{\sigma }_{\varepsilon }^{2}$ with the residual variance ${\sigma }_{\varepsilon }^{2}$. The residual $\varepsilon$ contains both genetic and non-genetic elements, from unidentified QTLs and within-trial error variation, respectively.

The second model, *IBD.SQM_F* can be expressed as:

$$Y = X\beta + M_{q} a_{q} + \varepsilon$$

$$a_{q} \sim { }N\left( {0,I_{P} \sigma_{q}^{2} } \right)$$

$$\varepsilon \sim { }N\left( {0, \oplus_{k = 1}^{F} I_{{n_{k} }} \sigma_{{\varepsilon_{k} }}^{2} } \right),$$

where the residual term $\varepsilon$ has family-specific VCOV structure written as ${\oplus }_{k=1}^{F}{I}_{{n}_{k}}{\sigma }_{{\varepsilon }_{k}}^{2}$, in which ${\sigma }_{{\varepsilon }_{k}}^{2}$ is the residual variance of the ${k^{th}}$ family whose family size is ${n}_{k}$ ($\sum_{k=1}^{F}{n}_{k}=N$). For the standard MAGIC design with a single family ($F=1$), IBD.SQM_U and IBD.SQM_F are equivalent models.

To account for QTLs elsewhere in the genome, i.e., genome background, we can add a set of cofactors to the model *QTL.SQM_F*, as in composite interval mapping (Zeng 1994; Jansen and Stam 1994), to obtain a multi-QTL model (MQM) called *IBD.MQM_F*, which is expressed as:

$$Y = X\beta + \mathop \sum \limits_{c \ne q} M_{c} a_{c} + M_{q} a_{q} + \varepsilon$$

$$a_{q} \sim N\left( {0,I_{P} \sigma_{q}^{2} } \right){\text{and }}a_{c} \sim N\left( {0,I_{P} \sigma_{c}^{2} } \right)$$

$$\varepsilon \sim { }N\left( {0, \oplus_{k = 1}^{F} I_{{n_{k} }} \sigma_{{\varepsilon_{k} }}^{2} } \right),$$

where the design matrix for a cofactor is ${M}_{c}$, and the column vector of random QTL effect at the cofactor is ${a}_{c}$, whose genetic variance is ${\sigma }_{c}^{2}$. ${M}_{c}$ and ${a}_{c}$ are structurally comparable to ${M}_{q}$ and ${a}_{q}$, but they represent different positions in the genome.

As an alternative to the inclusion of explicit cofactors, we can include a polygenic term, $g$, into the *IBD.SQM_F* model, leading to the *IBD.Kin_F* model:

$$Y = X\beta + M_{q} a_{q} + g + \varepsilon$$

$$a_{q} \sim { }N\left( {0,I_{P} \sigma_{q}^{2} } \right)$$

$$g\sim N\left( {0,K\sigma_{g}^{2} } \right)$$

$$\varepsilon \sim { }N\left( {0, \oplus_{k = 1}^{F} I_{{n_{k} }} \sigma_{{\varepsilon_{k} }}^{2} } \right),$$

where the VCOV structure of the polygenic term $g$ is $K{\sigma }_{g}^{2}$ in which ${\sigma }_{g}^{2}$ is the variance of the polygenic effect, and $K$ is a $N\times N$ kinship matrix based on genotype information on the whole genome using IBS information (VanRaden 2008). (We acknowledge that for consistency $K$ should have been based on IBD information. However, in practice we found little difference between kinship corrections based on IBD and IBS, and therefore, for convenience, decided to implement the widely used Van Raden software for calculation of kinship matrices.) To reduce the computational burden and avoid proximal contamination, we applied the leave-one-chromosome-out (LOCO) method for kinship matrix calculation (Yang et al. 2014).

The last model, *IBD.MQMkin_F*, combines cofactors, a polygenic term, and a residual term with family-specific VCOV structure:

$$Y = X\beta + \mathop \sum \limits_{c \ne q} M_{c} a_{c} + M_{q} a_{q} + g + \varepsilon$$

$$a_{q} \sim N\left( {0,I_{P} \sigma_{q}^{2} } \right)\;{\text{and}}\;a_{c} \sim N\left( {0,I_{P} \sigma_{c}^{2} } \right)$$

$$g\sim N\left( {0,K\sigma_{g}^{2} } \right)$$

$$\varepsilon \sim { }N\left( {0, \oplus_{k = 1}^{F} I_{{n_{k} }} \sigma_{{\varepsilon_{k} }}^{2} } \right).$$

The five IBD-based models are compared with a benchmark or reference model, a GWAS approach estimating fixed bi-allelic effects. This reference model is frequently employed in population-based mapping. We will refer to this model as *IBS.Kin*:

$$Y = X\beta + X_{q} \lambda_{q} + g + \varepsilon$$

$$g\sim N\left( {0,K\sigma_{g}^{2} } \right)$$

$$\varepsilon \sim N\left( {0, I_{N} \sigma_{\varepsilon }^{2} } \right),$$

where ${X}_{q}$ is a $N\times 1$ vector column whose elements are 0, 1, and 2, indicating numbers of allele copies based on IBS information, and ${\lambda }_{q}$ is the fixed bi-allelic effect at the QTL.

### QTL detection procedure

To identify QTLs with the above IBD-based mixed model approaches, we used a likelihood ratio test (LRT), $LRT=-2\left(\mathit{ln}\left(\frac{maxLo}{max{L}_{A}}\right)\right)$, comparing the likelihood under the alternative (${\sigma }_{a}^{2}\ne 0$) and null (${\sigma }_{a}^{2}=0$) hypotheses to evaluate variance components representing potential QTLs, with $max{L}_{0}$ and $max{L}_{A}$ being the Residual Maximum Likelihood (REML) (Gleeson and Cullis 1987) under null and alternative hypotheses estimated by ASReml-R (Version 3.0) (Butler et al. 2009). The LRT for the genetic variance (${\sigma }_{a}^{2}$) of a set of random QTL effects for a single QTL approximates a $0.5{\chi }_{0}^{2}+0.5{\chi }_{1}^{2}$ mixture distribution (Self and Liang 1987). The p-value corresponding to the LRT statistic was expressed as a -log10(p)). A simple multiple testing correction was performed via a Bonferroni threshold placed at a genome-wide significance level of 0.01. Around cofactors, we set an exclusion window of 20 cM within which no tests for further QTLs were performed. Genome scans for models with cofactors were repeated until the -log10(p) profile along the genome stabilized. For the reference GWAS model *IBS.Kin*, we utilized ASReml-R to incorporate the kinship matrix and performed a Wald test (Molenberghs and Verbeke 2007) to determine significant QTLs.

## Datasets

### Simulated MPP datasets

#### The motivation for simulating MPPs

By simulating QTLs with different segregation configurations in various types of MPP designs, we aim to test the performance of IBD-based models versus GWAS models, where we looked at mapping power and resolutions for major QTLs. In Section “Model performance assessment on simulated data”, we will define mapping power and resolution.

To create offspring populations with realistic marker profiles and segregation ratios, we based our simulations on four real Arabidopsis inbred lines with known genomes. These four lines served as parents in crosses that simulated diallel, NAM, and four-way MAGIC designs. With this number of parents, we still obtain enough details for insightful simulations, while computation time per simulation remains low. Genomes for progenies were simulated by implementing a crossover process in which progenies inherited markers and QTLs from parents following one of the MPP designs.

Phenotypes for the offspring individuals contained a contribution from alleles at three major QTLs, positioned on different chromosomes and at markers with varying genotypic configurations across the parents, to investigate the impact of the number of segregating families and allele frequencies on QTL detection. Details are given in Section “Details of the simulation”. Contributions of 24 minor QTLs were distributed across four chromosomes, including the three chromosomes with a major QTL to define a random polygenic effect. This polygenic effect was implicitly structured by family, depending on segregation or not in diallel and NAM families and allele frequency in MAGIC. The polygenic effect was further structured by the relations between the offspring individuals within families due to the transmission of QTL and marker alleles from parents to offspring. An independent error was added to major and minor QTL effects to determine heritability.

#### Details of the simulation

**Fig. 2:**
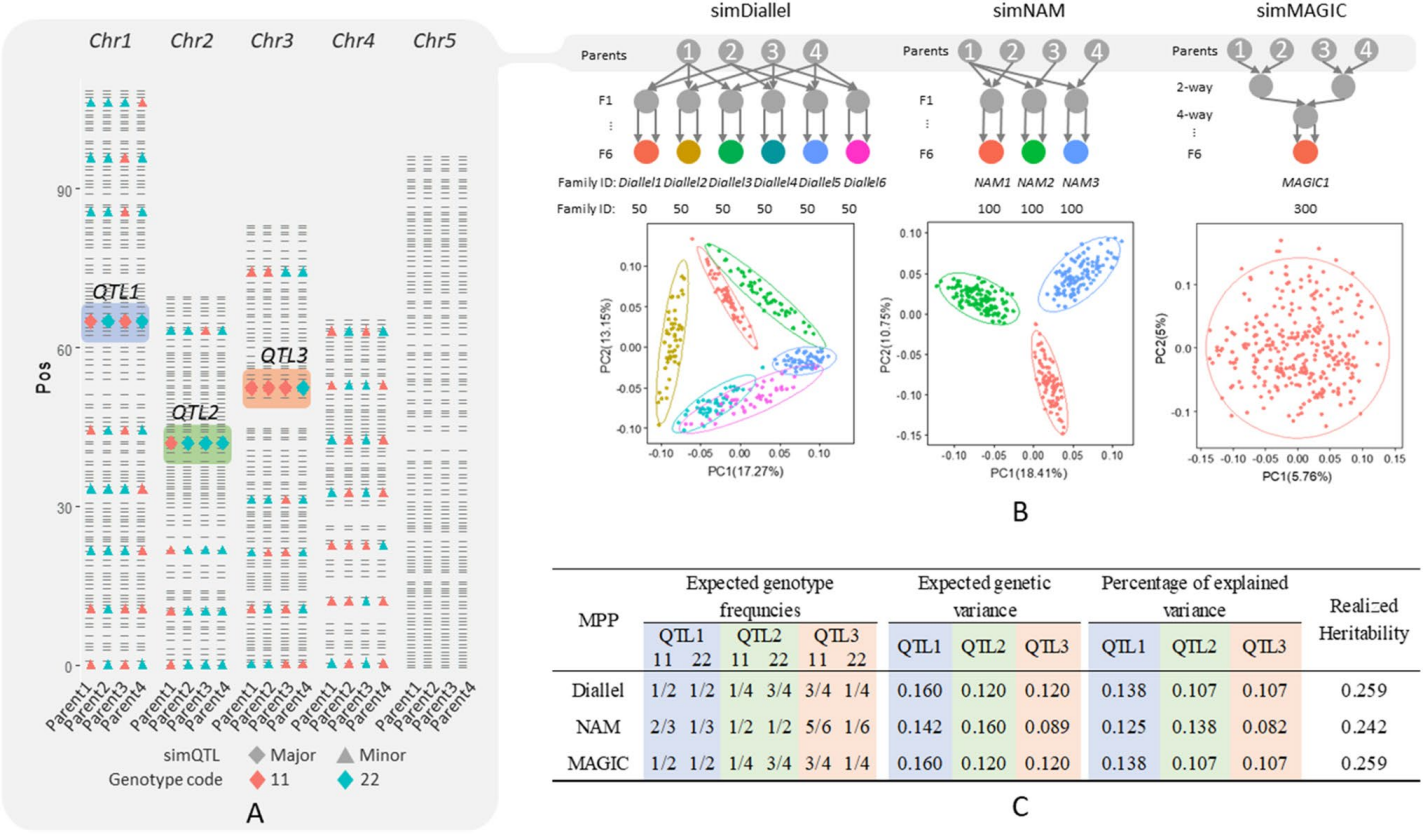
**A** Marker positions and genotypes for the four real inbred Arabidopsis genotypes used for simulating different MPP designs. Three major QTLs (diamonds) were simulated with an additive allelic substitution effect of 0.4 and the allele labeled as 1 increasing the trait; 24 minor QTLs (triangles) were simulated with the additive allelic substitution effect 0.1 with the allele labeled as 1 again increasing the trait. **B** Crossing schemes of simulated diallel, NAM, and MAGIC designs using the four parents with PCA plots for progenies based on simulated genome data. **C** Summary of expected genotype frequencies and genetic variance of each simulated major QTL and realized heritability of all major QTLs

Four inbred Arabidopsis lines with known marker genotypes were chosen as parents for making our simulated crosses: Bla-1 (*parent1*), Br-0 (*parent2*), Got-7 (*parent3*), and Kas-2 (*parent4*). These parents were randomly selected from the Arabidopsis *HapMap* collection (Baxter et al. 2010). More information is available at http://bergelson.uchicago.edu/?page_id=790. SNPs of parental lines were called against the reference sequence. A consensus linkage map with 462 markers was created from family-specific linkage maps for offspring populations involving the above parents. We selected positions at three markers, namely *simQTL1*, *simQTL2*, and *simQTL3*, on the consensus map to assign major QTL allelic effects. Marker genotypes in the parents were coded as 11 and 22 for homozygous reference and alternative alleles. For the three *simQTLs*, a homozygous genotype coded as 11 was carried by, respectively, two, one, and three parents (Fig. 2A). Starting at 20 cM above and below the major QTL position, minor QTLs were uniformly placed at distances of 10 cM from chromosomes 1 to 3. Chromosome 4 contained only minor QTLs. In total, 24 minor QTLs were placed at chromosomes 1 to 4. No QTLs were assigned to chromosome 5. This chromosome was used to assess the number of falsely discovered QTLs.

After deciding on simulated QTL positions, we simulated the genome of each progeny at the F6 generation from diallel, NAM, and MAGIC designs (Fig. 2B). Progeny was simulated from the parental genomes and crossing schemes using the tool RABBIT implemented in Mathematica (Zheng et al. 2015). Each chromosome in gametes in the generations from F1 to F6 was the random reshuffle of the unique parental genome due to crossovers. The number of crossovers on each chromosome followed a Poisson distribution, with the mean being the chromosome length in Morgan. The positions of crossovers were uniformly distributed over the chromosomes. In the F6 generation, we obtained realized genotypes at marker positions by adding missing genotypes at a rate of 5% randomly on the ‘true’ simulated marker genotypes. In practice, we expect this missingness rate to be lower, but we wanted an assessment of the robustness of our procedure.

The total population size for each MPP design was fixed at 300. This number is, first of all, realistic and allows sufficiently fast calculations for different MPP configurations while retaining sufficient power for QTL detection across the full population as well as within families. The six families in the diallel design, named *Diallel1* to *Diallel6*, contained 50 progenies per family, and the three NAM families, named *NAM1* to *NAM3*, included 100 progenies per family. For the MAGIC population, only one family, named *MAGIC1,* had 300 progenies. To show the genetic relatedness between progenies, we performed principal component analysis (PCA) on the ‘true’ simulated genome from each MPP design (Fig. 2B).

The phenotype of each progeny was the sum of the genetic effects of major and minor QTLs plus residual errors: $Y={az}_{1}+a{z}_{2}+a{z}_{3}+{\sum }_{i=1}^{24}b{z}_{i}+\varepsilon$, where $a$ and $b$ are the major and minor additive effects, respectively; and ${z}_{i}$ is the genotype indicator equal to 1 or -1 for the marker with homozygous genotype coded as 11 or 22, and equal to 0 for residual heterozygotes coded as 12 with very low frequencies in F6 generations; the residual term $\varepsilon$ followed a normal distribution $N(0, 1)$. For simplicity and convenience, we assigned only additive effects at the bi-allelic level to the simulated QTLs. To choose ‘realistic’ QTL effect sizes for major and minor QTLs, i.e., neither too low nor too high to compare the performance of the models, we did a grid search on QTL effect sizes. As an example, and for illustration in this paper, the effect size of major QTLs was taken to be 0.4, and the effect size of all minor QTLs was chosen to be 0.1. Supplementary Figure S1 shows an example of the distribution of simulated phenotypes in families of different MPP designs. We can calculate the expected genetic variance at the ${q}^{th}$ major QTL based on the formula $var(simQT{L}_{q})={a}^{2} \{E({Z}_{q}^{2} )-E{\left({Z}_{q} \right)}^{2}\}$ in simulated MPP designs with equal family sizes. The frequency of ${Z}_{q}$ relies on the specific MPP design. The percentage of explained variance is $var(simQT{L}_{q})/var\left(Y\right)$. The heritability of the simulated trait is a function of both major and multiple minor QTLs. We calculated the realized heritability from the genetic effect contributed by major QTLs’ additive effects over 500 replications (Fig. 2C).

#### Model performance assessment on simulated data

We simulated 500 replications for each MPP design and assessed the performance of all six models on these 500 simulated datasets. As criteria for performance, we chose mapping power and resolutions of major QTLs. The successful detection of a major QTL had to meet two requirements. Firstly, the −log10(p) value for the likelihood ratio test for the variance component of the QTL effects at that position should exceed the threshold of 4.2, obtained by the Bonferroni correction on all 462 markers on the consensus map at a genome-wide significance level of 0.01. Secondly, the distance between the true position of the simulated major QTL and the peak marker with the highest −log10(p) value on the same chromosome should be within the QTL window size (20 cM). The mapping resolution of a major QTL in our study was indicated by the average genetic distance between the true simulated position and the detected position with the highest -log10(p) value on the same chromosome over 500 runs. A shorter distance indicates a higher mapping resolution.

We compared our IBD-based mixed models to other approaches for genetic study in certain types of MPP designs. One of them is called ICIM (Li et al. 2007; Wang 2009) that is implemented in IciMapping (Meng et al. 2015) and GAPL (Zhang et al. 2019). Specifically, for the NAM design, we employed the joint inclusive composite interval mapping (JICIM) approach (Li et al. 2011) in the software IciMapping. For the MAGIC design, we used the *PLQ* function in the software GAPL for background-controlled QTL mapping (Zhang et al. 2017). However, the ICIM-based approaches cannot be applied to diallel designs. Another package for QTL mapping in MPP designs is mppR (https://cran.r-project.org/web/packages/mppR/index.html). The MQE model of mppR defines QTL effects for genomic positions at di-allelic, parental, and ancestral levels and chooses the type of QTL effect that produces the highest significance (Garin et al. 2017). The MQE model can be applied to diallel and NAM designs but cannot handle MAGIC designs. QTL mapping for simulated NAM and MAGIC designs was performed using IciMapping and GAPL by setting the mapping method ICIM-ADD to estimate only additive effects with a LOD threshold of 4.2, which was the Bonferroni-corrected threshold that we used in our mixed model approach. Equally so, for QTL mapping in simulated diallel and NAM designs by mppR, we applied a threshold of 4.2 to map QTLs with different effect types using the MQE mapping model. QTL mapping power and resolution for alternative software packages were summarized with the same QTL window size of 20 cM as we used in our mixed models.

### Empirical MPP designs

Besides the three simulated MPP designs, we re-analyzed empirical diallel, NAM, and MAGIC designs collected from previous studies and the vegetable breeding company Rijk Zwaan. Six datasets are summarized in Table 2. Given the available genotypic and phenotypic information and the consensus linkage map from previous studies, we calculated IBDs using RABBIT and mapped QTLs by the IBD-based mixed models using ASReml-R following the framework described in Section “Methodology”.

**Table 2:**
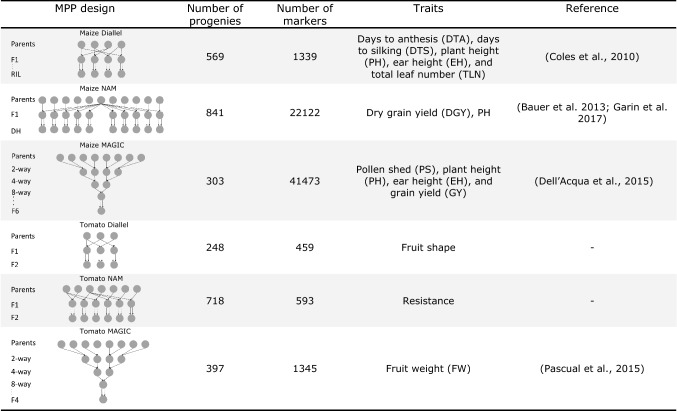
Summary of empirical maize and tomato datasets of diallel, NAM, and MAGIC designs collected from previous studies

Following Verbyla (2019), for each combination of data and model, we calculated the Bayesian information criterion (BIC) to identify empirically an appropriate mixed model: $BIC=\left(D{F}_{fixed}+D{F}_{var}\right)\times ln\left(n-r+D{F}_{fixed}\right)-2ln({L}_{max})$, where *n* is the number of observations (population size) and *r* is the rank of the fixed effects design matrix (related to the number of families); $D{F}_{fixed}$ and $D{F}_{var}$ are the degree of freedoms for fixed (families) and random parameters (QTL effect variances), respectively; ${L}_{max}$ is the estimated residual maximum likelihood. We used the *infoCriteria *function from the package asremlPlus (Brien 2021) with the by-default setting IClikelihood = “REML” to calculate the BIC. Parental effects at QTL candidates were estimated from the model with the smallest BIC value.

#### Maize MPP designs

In the first maize diallel design, recombinant inbred lines (RILs) were derived from crosses between four inbred parents (Coles et al. 2010). The four parents represent distinct germplasm groups in temperate (B73 and B97) and tropical (CML254 and Ki14) types of maize (Liu et al. 2003), and parents of different types were crossed with each other. In total, 569 progenies in four families were obtained with 1,339 genotyped markers. Traits of interest were days to anthesis (DTA), days to silking (DTS), plant height (PH), ear height (EH), and total leaf number (TLN) measured in long-day and short-day environments. The photoperiodic responses of those traits were the difference between long-day and short-day responses. The previous study applied the package MCQTL to perform QTL mapping in joint and separate families (Jourjon et al. 2005).

The second maize MPP design was the Dent panel of the EU-NAM population. Ten families of 841 DH progenies were derived from 11 parents in which the central parent F353 was crossed with ten peripheral lines (Bauer et al. 2013). We used the consensus map of 22,122 Panzea markers from previous studies (Giraud et al. 2014; Bustos-Korts et al. 2016; Garin et al. 2017). Markers were removed when one or more parents had missing genotype information, which led to 15,813 markers for the IBD calculation and QTL analysis. QTL mapping for DGY and PH was previously performed in MQE model that allowed mixture types of QTL effects at parental, ancestral, and bi-allelic levels (Garin et al. 2017).

The last maize MPP design is the eight-way MAGIC population (Dell’Acqua et al. 2015). The eight maize inbred lines (A632, B73, B96, F7, H99, HP301, Mo17, and W153R) were crossed in the format of 35 independent breeding funnels containing two-way, four-way, and eight-way crosses. According to the previous study (Dell’Acqua et al. 2015), the two-way cross $B96\times HP301$ failed during the MAGIC population construction. A ninth parent (CLM91) was introduced in the two-way cross $B73\times CML91$ to complement four-way crosses with failed two-way cross $B96\times HP301$. Each funnel was advanced by single seed descent to the F6 generation with 529 progenies and 41,473 genotyped markers. 529 progenies were phenotyped in two different environments for days to pollen shed (PS), plant height (PH), ear height (EH), and grain yield (GY). In the previous study, two methods were applied for QTL mapping (Dell’Acqua et al. 2015): the linkage mapping approach using genotype probabilities as predictors and imposing a kinship VCOV structure on the polygenic term, and the association mapping approach estimating allelic additive effects. However, our study utilized only 303 progenies derived from the initial eight parents after excluding progenies derived from the ninth parent (CLM91), because the pedigree information related to the ninth parent was not available for us to calculate IBDs.

For these empirical maize MPP designs, we did not use all the markers described above for analysis. Instead, we selected the markers at each 0.5 cM for IBD computation and QTL mapping to remove colocated markers and speed up the analysis.

#### Tomato MPP designs

We had two tomato MPP designs provided by the breeding company Rijk Zwaan. The first one is a diallel F2 design constructed by crossing three inbred lines differing in fruit shape. The three inbred parents were crossed with each other to generate 248 F2 progenies with 459 genotyped markers. The second tomato MPP design combines two NAM F2 designs, where the two central parents are connected. We call the whole setup a connected NAM design where 718 progenies were genotyped with 593 markers and phenotyped based on their resistance level.

The last tomato MPP design is an eight-way MAGIC population. In this population, eight inbred tomato lines with different molecular and physiological levels were selected as founders to capture the wide genetic diversity (Pascual et al. 2013). In the previous research, the trait of interest for QTL mapping was fruit weight (FW) measured in two locations for 397 F4 individuals with 1345 genotyped markers (Pascual et al. 2015). Interval mapping with adjusted P-values and GWAS approaches were applied for the QTL analysis. For the interval mapping, the previous study implemented R package mpMAP to estimate parental effects based on multipoint probabilities (Huang and George 2011). The percentage of phenotypic variation was calculated by fitting all significant QTLs in a full mixed model (Huang and George 2011; Pascual et al. 2015). For the GWAS approach, the kinship matrix was computed to describe the VCOV structure of the polygenic term in a mixed linear model using software TASSEL (Bradbury et al. 2007).

## Results

### Results of simulated MPP designs

#### MPP simulation

To illustrate relatedness in each simulated MPP design, we performed PCA on progenies using the simulated genome (IBS) (Fig. 2B). The PCA plots of diallel and NAM designs show apparent family clusters. For the simulated MAGIC design, no visible family clusters were present.

Parental origins can be uncertain at multiple positions with non-segregating markers or missing genotypes. Using the pedigree information and genomes of parents and offspring, we reconstructed the parental origins represented by IBDs. We extracted the highest parental IBD probabilities at each marker position per progeny and averaged the maximum IBD probabilities of this position across all progenies. The averaged maximum IBD probabilities at all positions were above 0.9 in all simulated MPP designs demonstrating the high quality of the parental genomic reconstructions in the offspring populations.

#### Model performance assessment

**Fig. 3:**
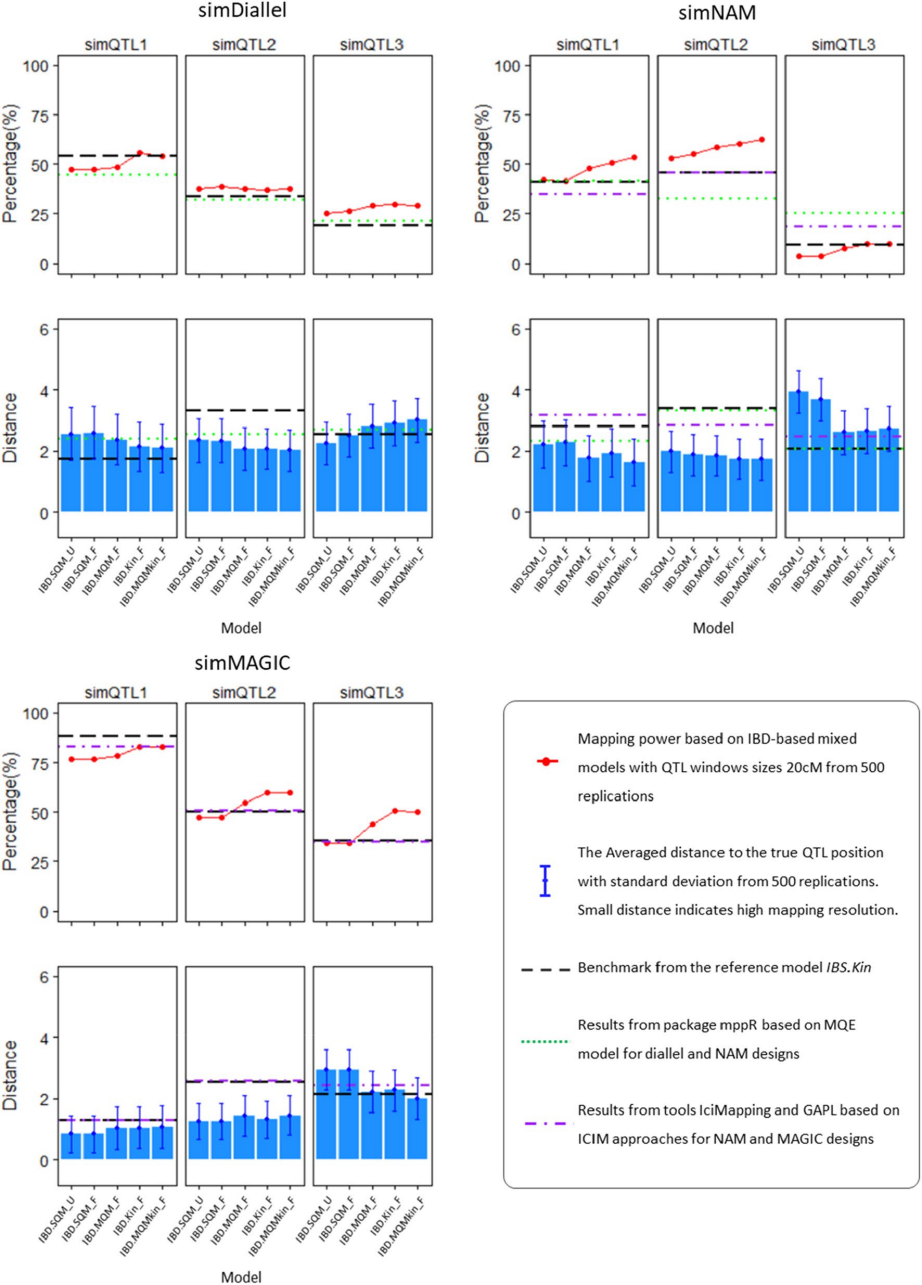
The model performance assessment is based on simulated MPP designs in terms of mapping power (**upper panel**) and mapping resolution (**lower panel**). IBD-based mixed models are compared with the multiple QTL (MQE) model in the mppR package for simDiallel and simNAM designs, and ICIM-based models implementing IciMapping and GAPL tools for respective simNAM and simMAGIC designs

By comparing the QTL mapping results of the six models on simulated MPP designs, we evaluated the efficiency of using IBD information for genetic predictors and the validity of using the mixed model approach to account for the multilevel relatedness of offspring within and across families. Figure 3 demonstrates the performance of each model in terms of mapping power and resolutions at the three major QTLs in each MPP design. Moving from basic to advanced IBD-based mixed models, we can observe the increasing trend regarding mapping power and resolution for major QTLs. On chromosome 5 where neither major nor minor QTLs were simulated, we counted how many markers were detected as QTLs (i.e., false positives) by each of the six models over 500 runs in each MPP design. Given 110 markers on chromosome 5, we expected the number of false QTLs over 500 runs using a threshold of 4.2 to be $110\times 500\times {10}^{-4.2}=3.47$. The reference model *IBS.Kin* identified 2, 3, and 5 positions as false QTLs in the respective simulated diallel, NAM, and MAGIC designs; among IBD-based models, only the advanced model *IBD.MQMkin_F* detected one false QTL on chromosome 5 in the simulated diallel design over 500 runs. The mapping tools ICIMapping and GAPL detected 3 and 4 false QTLs on chromosome 5 in simulated NAM and MAGIC designs, respectively, while with mppR, 5 and 2 false QTLs were detected on chromosome 5 in simulated NAM and diallel. Therefore, no large deviations from the expected number of false positives were observed for the alternative mapping methods either.

In the simulated diallel design, the IBD-based models *IBD.Kin_F* and *IBD.MQMkin_F,* incorporating polygenic effects, generally performed better than other models considering both mapping power and resolution. For the *simQTL1* segregating in four out of six families, the mapping power by *IBD.MQMkin_F* was higher than that at *simQTL2* and *simQTL3*, both segregating in three out six families.

In the simulated NAM design, IBD-based models have advantages over the reference model *IBS.Kin* in improving mapping power and resolution for *simQTL1* and *simQTL2*. Notably, the advanced IBD-based model *IBD.MQMkin_F* detected *simQTL2*, which is segregating in all three families, with the highest mapping power and resolution. For *simQTL1,* segregating in two out of three families, mapping power and resolution obtained from *IBD.MQMkin_F* were also higher than other models. However, for *simQTL3* that segregated in only one family, *IBD.MQMkin_F* detected this QTL with slightly higher mapping power than other IBD-based models but the mapping resolution was lower than the *IBS.Kin* model.

The simulated MAGIC design seems to be the most promising MPP for detecting all three *simQTLs*—all of them were detected with relatively high mapping power and resolutions over diallel and NAM designs. Especially for *simQTL1* with an expected genotype frequency of 0.5, both the advanced IBD-based model *IBD.MQMkin_F* and the reference IBS-based model *IBS.Kin* successfully detected this QTL with high mapping power and resolution. For *simQTL2* and *simQTL3*, advanced models *IBD.Kin_F* and *IBD.MQMkin_F* performed better than the rest of the models considering both mapping power and resolutions.

When comparing our IBD-based mixed model approach to alternative approaches from the literature, we observed (see Fig. 3) that results of *IBD.MQMkin_F* were comparable to ICIM-based approaches for simulated NAM and MAGIC designs, while the same *IBD.MQMkin_F* was comparable to the MQE model for simulated diallel and NAM designs.

In the simulated diallel design, the mapping power of *simQTL1* and *simQTL3* by *IBD.MQMkin_F* were higher than the MQE model using the mppR package, while no apparent difference occurred between *IBD.MQMkin_F* and MQE for *simQTL2* in terms of mapping power. For the mapping resolution, *IBD.MQMkin_F* and other IBD-based models performed better than the MQE model on all *simQTLs*.

In the simulated NAM design, we can compare *IBD.MQM_F* with JICIM approach in the software IciMappig and MQE model in the mppR. For *simQTL1* and *simQTL2*, *IBD.MQM_F* performed better than both JICIM and MQE models with higher mapping power and resolution. However, for *simQTL3* that segregated in only one of three families, *IBD.MQMkin_F* detected this QTL with lower mapping power and resolution than JICIM and MQE, because of the test for a variance component being underpowered for a single biparental population.

For the simulated MAGIC design, we see that *IBD.MQMkin_F* detected *simQTL2* and *simQTL3* with higher mapping power and resolution than the ICIM-based approach in the GAPL software. Both approaches detected *simQTL1* with equally high mapping power, and the mapping resolution is slightly higher by using *IBD.MQMkin_F* than GAPL.

### Results of empirical MPP designs

#### Maize diallel design

**Fig. 4:**
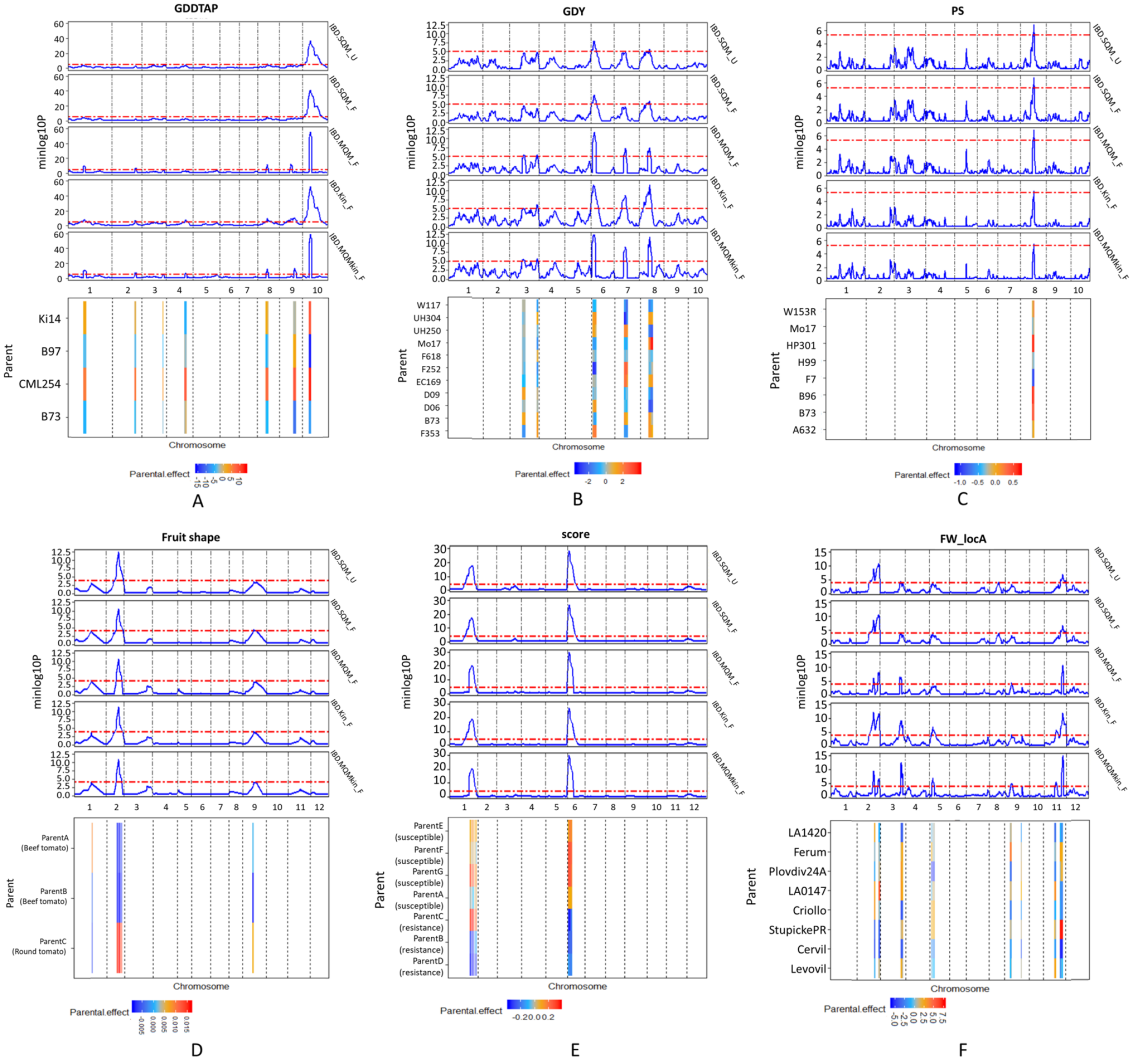
Mapping results of some selected traits as examples in the empirical MPP designs: **A** maize diallel, **B** maize NAM, **C** maize MAGIC, **D** tomato diallel, **E** tomato NAM, and **F** tomato MAGIC using the five IBD-based mixed models. **Upper panel** QTL profiles from the five IBD-based mixed model approaches. **Lower panel** Estimation of parental effects at QTLs detected by a model selected with the smallest BIC among the five models. The BIC values of other models and mapping results of other traits are provided in Supplementary Table S1

The maize diallel design generated four families from four parents with multiple traits measured and analyzed in the previous study (Coles et al. 2010). In this research, we re-analyzed the photoperiodic responses of DTA, DTS, the difference in GDD between DTA and DTS (GDDASI), PH, EH, and TLN (Fig. 4A; Supplementary Figure S2). The analysis of those traits using IBD-based models showed multiple shared QTLs, but the last model, *IBD.MQMkin_F* is superior to other models because it detected most QTLs with increased mapping signal and the relatively small BIC value (Supplementary Table S1).

Coles et al. (2010) reported QTL mapping using separate biparental families and joint families and found that joint mapping detected more QTLs with higher resolution. These QTLs were found to coincide with key flowering time QTLs on chromosomes 1, 8, 9, and 10. Here we compare our results to the joint mapping of Coles et al. (2010): In the example of trait GDDTAP, we detected 7 QTLs on chromosomes 1, 2, 3, 4, 8, 9, and 10, which is comparable to the 6 QTLs detected on chromosomes 1, 2, 4, 8, 9, and 10 in the joint model by Coles et al. (2010). For other traits (Supplementary Figure S2), the advanced model *IBD.MQMkin_F* could detect most reported QTLs in the study by Coles et al. (2010). Due to the smaller BIC of the *IBD.MQMkin_F* model with more detected QTLs (Supplementary Table S1), we fitted these 7 detected QTLs in this advanced IBD-based mixed model to estimate the parental effects at those QTLs for trait GDDTAP. It shows that the parents of temperate type (B73 and B97) contribute negative effects at those QTLs while the other two parents of tropical type (CML254 and Ki14) contribute the positive effects at most of those detected QTLs.

#### Maize NAM design

In the maize NAM population, we identified two QTLs for DGY on chromosomes 6 and 8 using models without correction for genomic background, i.e., *IBD.SQM_U* and *IBD.SQM_F* (Fig. 4B; Supplementary Figure S3). Including either cofactors, the polygenic term, or both increased the magnitude of the mapping signals for the two QTLs and allowed us to detect new QTLs on chromosomes 3 and 7 for trait DGY. As for the mapping results of PH, the advanced models *IBD.kin_F* and *IBD.MQMkin_F* detected a new QTL on chromosome 5 and increased the magnitude of mapping signals for other QTLs that were also detected by basic models.

For both DGY and PH, our models detected QTLs that were detected by Garin et al. (2017) using the MQE model that combined genome-wide scans at bi-allelic, parental, and ancestral levels. Our study adopted a stringent threshold via Bonferroni correction, so some QTLs with relatively weaker signals were missed compared to the analysis by (Garin et al. 2017).

#### Maize MAGIC design

For the maize MAGIC population, we mapped the QTLs for trait PS, PH, EH, and GY (Fig. 4C; Supplementary Figure S4). Using all five models, we detected one QTL on chromosome 8 for PS, and identified one QTL on chromosome 6 for both traits PH and GY, while no QTL was detected for EH, so the mapping profiles for EH were not shown. Dell’Acqua et al. (2015) used all 529 magic maize progenies for QTL mapping, but we selected 303 progenies individuals in the population derived from the initial eight parents. We could confirm some major QTLs detected earlier by Dell’Acqua et al. (2015) in their analysis, but due to the smaller total population size in our analysis, we missed some QTLs, e.g., two QTLs for trait EH, one QTL on chromosome 8 for PH and PS.

#### Tomato diallel design

The diallel F2 design of two beef tomatoes and one round tomato generated a population of diverse fruit shapes, and we identified some QTLs for fruit shape (Fig. 4D). In total, we detected three QTLs on chromosomes 1, 2, and 9. All three QTLs were detected using *IBD.SQM_F* and *IBD.MQMkin_F* models, while other models missed the QTL on chromosome 1 or 9 with relatively weak signals. The three detected QTLs using were fitted in the model with the smallest BIC value (*IBD.MQMkin_F)* to estimate the parental effects (Supplementary Table S1). It shows that the parental effect of one beef type tomato (parent B) contributed negatively to the fruit shape at all three QTLs, and the other beef type tomato (parent A) show negative effects at two QTLs on chromosomes 2 and 9 on which the round type tomato (parent C) show positive effects at these two QTLs.

#### Tomato NAM design

In the connected NAM F2 design, a resistant parent crossed with four susceptible lines, and one of the susceptible lines was crossed with another two resistant lines. All five IBD-based mixed models identified two QTLs with strong signals on chromosomes 1 and 6 (Fig. 4E). Because all models detected those two QTLs with strong signals, the parental effects estimated by the five models show no big difference. As an example, we estimated the parental effect in the *IBD.MQMkin_F* model to show that the three resistant parents (parent C, B, and D), at the strongest QTL on chromosome 6, contributed negatively to the disease score. Tomato breeders have successfully fine-mapped this QTL as a strong resistance gene.

#### Tomato MAGIC design

In the eight-way MAGIC F4 population, we mapped QTLs underlying fruit weight measured at two locations that we will refer to as A and B. The model *IBD.MQMkin_F* detected most QTLs for location A, and all models detected three consistent QTLs for location B (Fig. 4F; Supplementary Figure S5). For location A, models that accounted for the genomic background by adding either cofactors or the polygenic term or both (*IBD.MQM_F*, *IBD.Kin_F*, or *IBD.MQMkin_F*), compared to *IBD.SQM_U* and *IBD.SQM_F*, allowed us to detect more QTLs on chromosomes 5 and 9. *IBD.Kin_F* and *IBD.MQMkin_F* identified two linked QTLs on chromosomes 2 and 11. The advanced model *IBD.MQMkin_F*, among the five models, has the smallest BIC value (Supplementary Table S1).

The previous study by (Pascual et al. 2015) applied two approaches for QTL detection in this design. One was the interval mapping for founder effects based on the multipoint probability calculations, and another was a GWAS approach incorporating a polygenic term. Pascual et al. (2015) analyzed fruit weight measured at location A using interval mapping and detected nine QTLs on chromosomes 2, 3, 5, 7, 8, and 11. In our study, we detected eight QTLs with the *IBD.MQMkin_F* model. We fitted QTLs in the model *IBD.MQMkin* to estimate the percentage of phenotypic variation explained by the QTLs. The eight QTLs identified in our study slightly increased the explained percentage of phenotypic variation, from 51% in Pascual et al. (2015) to 56% now. For the fruit weight measured at location B, we detected the three QTLs on chromosomes 2, 3, and 11 in the same region that has been previously detected using interval mapping (Pascual et al. 2015). The explained percentage of phenotypic variation of 33% was close to 34% in the previous study. The parental effects on those QTLs are estimated from the *IBD.MQMkin_F* model can be conformed with parental performance. For instance, the parental effects at all detected QTLs of FW showed that parent *Cervil*, with the lightest fruit, contributed negative values to fruit weight in both A and B locations.

## Discussion

### MPPs show design-specific properties

Different MPPs are constructed for different goals. A diallel mating design of carrot was constructed to dissect the genetic architecture of shoot growth by estimating the general and specific combing abilities and non-additive effects (Turner et al. 2018); a maize NAM population was proved to be able to capture small effect QTLs when they were shared by families (Ogut et al. 2015); MAGIC populations allow a large set of QTLs segregating with higher resolution and thus can increase the chance of detecting QTLs (Mackay et al. 2014). Another study compared the different designs of biparental, multiparental, and association panels in the context of the genome sequencing era to show their complementarity in genetic studies (Pascual et al. 2016). Our study simulated diallel, NAM, and MAGIC designs using four real Arabidopsis inbred lines. We focused on those MPP designs because they are often used in genetic research and breeding programs, and NAM and MAGIC designs are components of other more general designs that can also be analyzed using our framework.

QTL mapping results are impacted by the crossing schemes between parents in MPP designs and the genomic background. Probabilities for segregation differ between families owing to the specific crosses between parents. For *simQTL3* segregating in only one family of the simulated NAM design, even the advanced IBD-based models could not remarkably improve the mapping results, whereas this QTL could be detected with higher mapping power and resolution in both diallel and MAGIC designs. The reason might be that the joint family QTL mapping in NAM designs favors the large-effect QTLs or QTLs shared by most families (Ogut et al. 2015; Bajgain et al. 2016; Garin et al. 2017). *simQTL2* and *simQTL3* were expected to have the same allele frequency in the simulated MAGIC or diallel design, but it turned out that *simQTL2* could be detected with higher mapping power and resolution than *simQTL3*. The reason is that chromosome 2, where *simQTL2* is located, provided a more contrasting genomic background combining *parent1* with other parents than chromosome 3. The problem for the IBD-based mixed models with *simQTL3* in the NAM MPP was that this QTL segregated in one biparental cross only and the test for a variance component related to a QTL will be underpowered when the QTL represents too few allelic effects (Crainiceanu and Ruppert 2004). In such cases, a conventional Wald test for a fixed QTL substitution effect would have been more adequate.

We expect multilevel relatedness between individuals as being full-sib, half-sib, or unrelated depending on the specific MPP design. In the simulated MAGIC design, each progeny's genome was the uniformly reshuffled genome of all parents (Pascual et al. 2015; Dell’Acqua et al. 2015; Ongom and Ejeta 2018), and thus, no apparent clustering or grouping was observed in the PCA plot. In simulated diallel and NAM design, we observed multilevel relatedness of offspring within and across families: The full-sib progenies gathering in their family clusters were more genetically correlated than the half-sib progenies sharing one common parent.

Different MPP designs require different QTL analysis models with the first question being the choice of using IBS or IBD information in the genetic predictors and a second question concerning how to deal with individual relatedness within and across families.

### The IBD is informative to reflect the genome origins

We used the observed IBS with 5% of missing genotypes to estimate IBD probabilities and then compared IBS-based with IBD-based models. Parental origins can be ambiguous at non-segregating, missing, or mis-genotyped loci based on IBS information, whereas IBD probabilities inferred by the pedigree information and the whole genome can reduce uncertainty for genome origins (Zheng et al. 2015, 2018). Generally, IBD-based models were more effective than the IBS-based model (*IBS.Kin*) concerning mapping power and resolutions for major QTLs.

A reliable approach for precise IBD computations is fundamental for inferring parental origins and performing IBD-based QTL mapping, but only a few methodologies are available, and most of them were limited to specific MPP designs (Verbyla et al. 2014; Broman et al. 2018). In this study, we used a general hidden Markov model framework to construct parental origins (Zheng et al. 2015, 2018), which was successfully extended to all kinds of MPP designs.

IBD information is not only useful as a basis for genetic predictors in the QTL mapping models but also valuable in consensus map construction. The traditional process of consensus map construction can be tedious in the MPP context, including marker cleaning, grouping, ordering (Wu et al. 2008; Taylor 2018), and map integrating (Endelman and Plomion 2014). In future work, we can utilize IBD probabilities to infer the recombination between markers for genetic map construction (Zheng et al. 2019), as thus, we simplify and optimize the MPP analysis framework from the beginning.

### Modeling family-specific VCOV structure on the residual term is recommendatory

Owing to the major and minor QTLs with varying segregation configurations in each family, the family-specific distribution of phenotypes motivated us to model a family-specific VCOV structure on the residual term to account for the family genetic background. However, based on the simulated diallel and NAM designs, there is no substantial evidence to show the advantage of using *IBD.SQM_F* model over *IBD.SQM_U* model. Another study also reports that modeling a heterogeneous VCOV structure on the residual term may not always improve the mapping results (Garin et al. 2017). One of the reasons might be the limited family size, e.g., a family size of 50 or 100 in simulated diallel or NAM designs may not have been big enough to reveal the heterogeneity of variance components between families. Still, modeling a heterogeneous error can be advantageous in the fitting of single-locus QTL models in initial genome scans, whereas in the latter stages of the building of a multilocus QTL model, the advantage of a heterogeneous error diminishes because most of the genetic effects have been incorporated in the QTL structure leaving the residual less heterogeneous.

Non-genetic factors can also cause family-specific variation. It is common in an MPP breeding program where each family is separately established and subjectively phenotyped by different breeders in separate locations. Therefore, we recommend modeling a family-specific VCOV structure on the residual term to account for the potential family background due to both genetic and non-genetic reasons. For choosing an appropriate VCOV model for a trait in a particular MPP, we proposed a model selection procedure based on BIC.

### Imposing a kinship structure on the polygenic effect accounts for individual relatedness

Including only significant positions as cofactors from the initial genome-wide scans can lead to ignoring part of the genetic variance and missing heritability (Myles et al. 2009). To deal with smaller QTLs that may go unnoticed, we incorporated a polygenic effect whose VCOV structure is described by a kinship matrix. A study on a three-way barley cross has shown that the inclusion of the kinship VCOV structure containing co-ancestry information can avoid unrealistic marker–trait associations (Malosetti et al. 2011). In our study, the re-analysis of the empirical tomato MAGIC population with the polygenic term allowed us to detect more QTLs for fruit weight measured in location A with the relatively small BIC. In the simulated MAGIC design, adding a polygenic term (*IBD.Kin_F*) increased mapping power and resolutions for all *simQTLs* compared with *IBD.SQM_F* and *IBD.MQM_F*.

Population-based mapping approaches incorporating individual relatedness are widely applied to association mapping panels. In specific MPP designs (e.g., diallel and NAM), multilevel relatedness exists between individuals as being full-sib, half-sib, and unrelated within and across families. A priori no population structure is expected in standard MAGIC designs, but MAGIC lines may still show complicated realized genetic relationships. Multilevel relatedness can be corrected by using a general QK model where the Q matrix accounts for family structure, and the pairwise relationship matrix K deals with the individual relatedness (Yu et al. 2006). Likewise, our study modeled the family-specific residual term to correct for family structure and imposed a kinship VCOV structure on the polygenic term to incorporate multilevel relatedness.

### The advanced IBD-based model works well for general MPP designs

To sum up, we can refer to conceptions from family-based and population-based mapping approaches to explain the efficiency of our approach. Family-based QTL mapping assumes QTL effects to be multiallelic and referring to parental origins. For the estimation of parental effects at the QTL, we need design matrices that are functions of IBD probabilities. Popular population-based mapping strategies employ the mixed model approach to deal with multilevel relatedness by imposing family-specific and kinship-based VCOV structures on the non-genetic residual and the polygenic term, respectively. Family-based and population-based mapping approaches complement each other, and their synthesis in an advanced IBD-based mixed model approach (*IBD.MQMkin_F*) offers us a robust and comprehensive solution to map QTLs in general MPP designs. In our simulation study, we observed no case where the *IBD.MQMkin_F* model performed significantly worse than other IBD-based models in terms of mapping power and resolution, and this model is also competitive with other tools developed for specific MPP designs. Most results from empirical MPP designs also show that the unified *IBD.MQMkin_F* model detected most QTLs with relatively small BIC, and the major QTLs were comparable to those identified by previous studies.

## Supplementary Information

Below is the link to the electronic supplementary material.

Supplementary file1 (DOCX 2744 KB)

Supplementary file2 (DOCX 20 KB)
