# Supplementary material for: An IBD-based mixed model approach for QTL mapping in multiparental populations
Source: Theor Appl Genet. 2021 Aug 3;134(11):3643–60. doi: 10.1007/s00122-021-03919-7 (PMC8519866; doi:10.1007/s00122-021-03919-7)

Figure S1 An example taken out of a set of 500 replications demonstrates the family-specific phenotype distributions in simulated diallel, NAM, and MAGIC designs.

Figure S2 Mapping results for the empirical maize diallel design using the five IBD-based mixed models. **Upper panel** QTL profiles from the five IBD-based mixed model approaches. **Lower panel** Estimation of parental effects at QTLs detected by a model selected with the smallest BIC among the five models.

Figure S3 Mapping results for the empirical maize NAM using the five IBD-based mixed models. **Upper panel** QTL profiles from the five IBD-based mixed model approaches. **Lower panel** Estimation of parental effects at QTLs detected by the model with the smallest BIC among the five models.

Figure S4 Mapping results for the empirical maize MAGIC using the five IBD-based mixed models. **Upper panel** QTL profiles from the five IBD-based mixed model approaches. **Lower panel** Estimation of parental effects at QTLs detected by the model with the smallest BIC among the five models.

Figure S5 Mapping results for the empirical tomato MAGIC design using the five IBD-based mixed models. **Upper panel** QTL profiles from the five IBD-based mixed model approaches. **Lower panel** Estimation of parental effects at QTLs detected by the model with the smallest BIC among the five models.

Figure S1


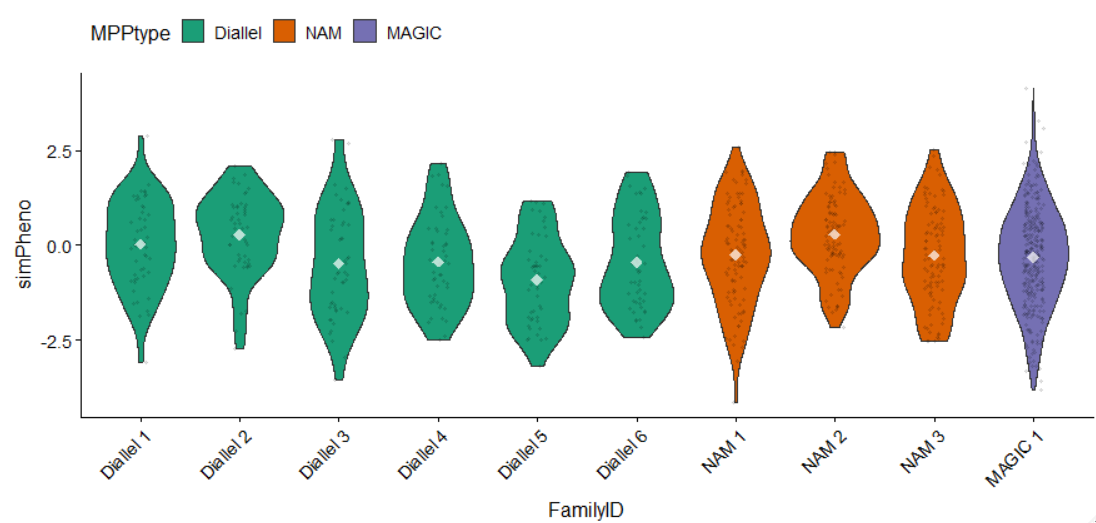


Figure S2


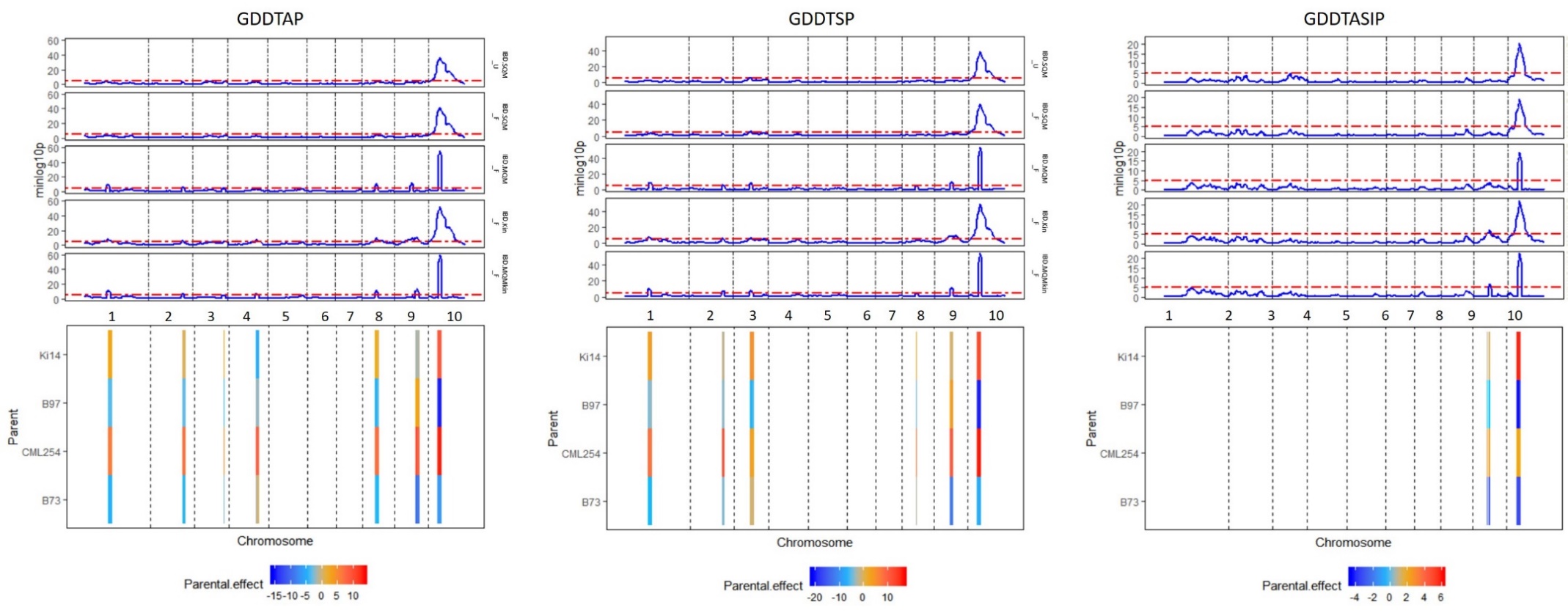


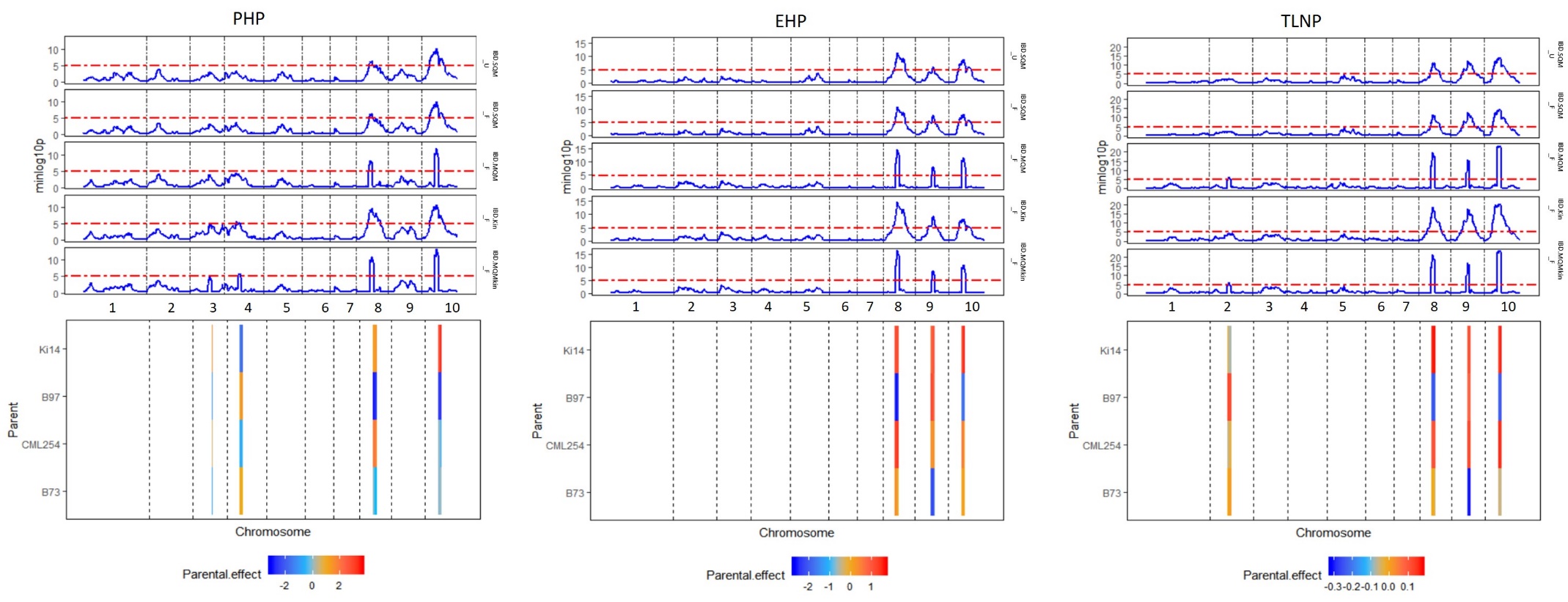


Figure S3


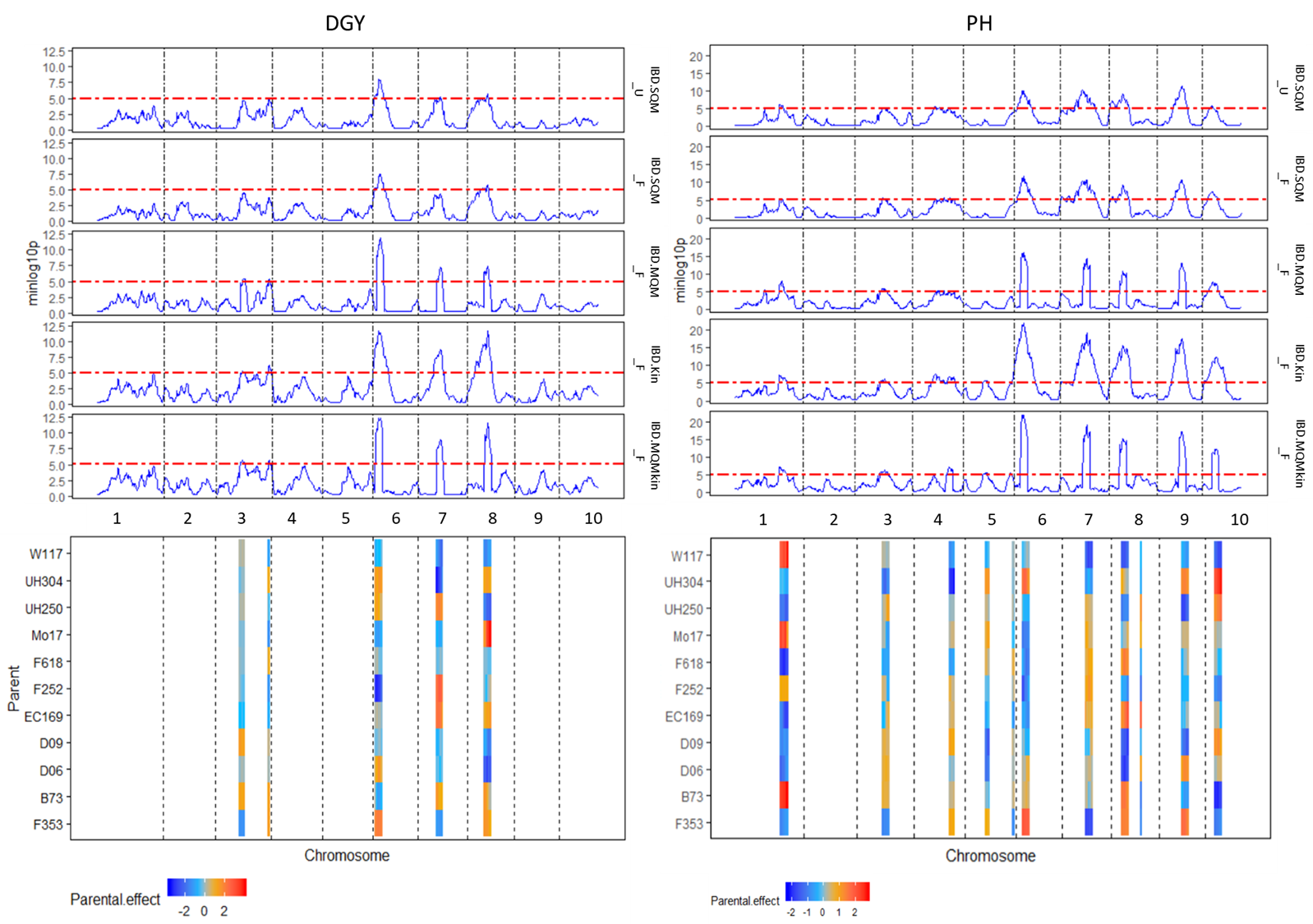


Figure S4


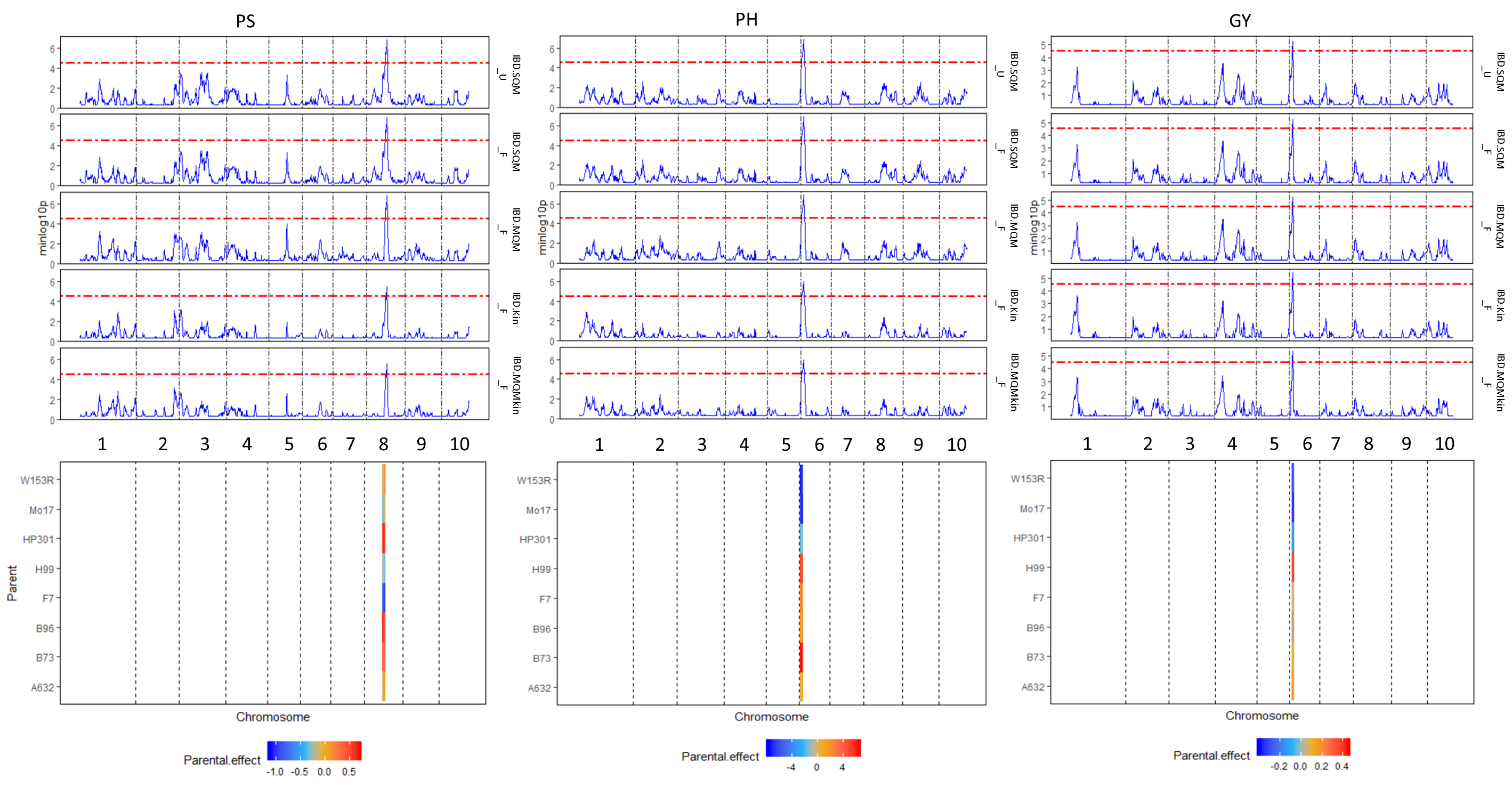


Figure S5


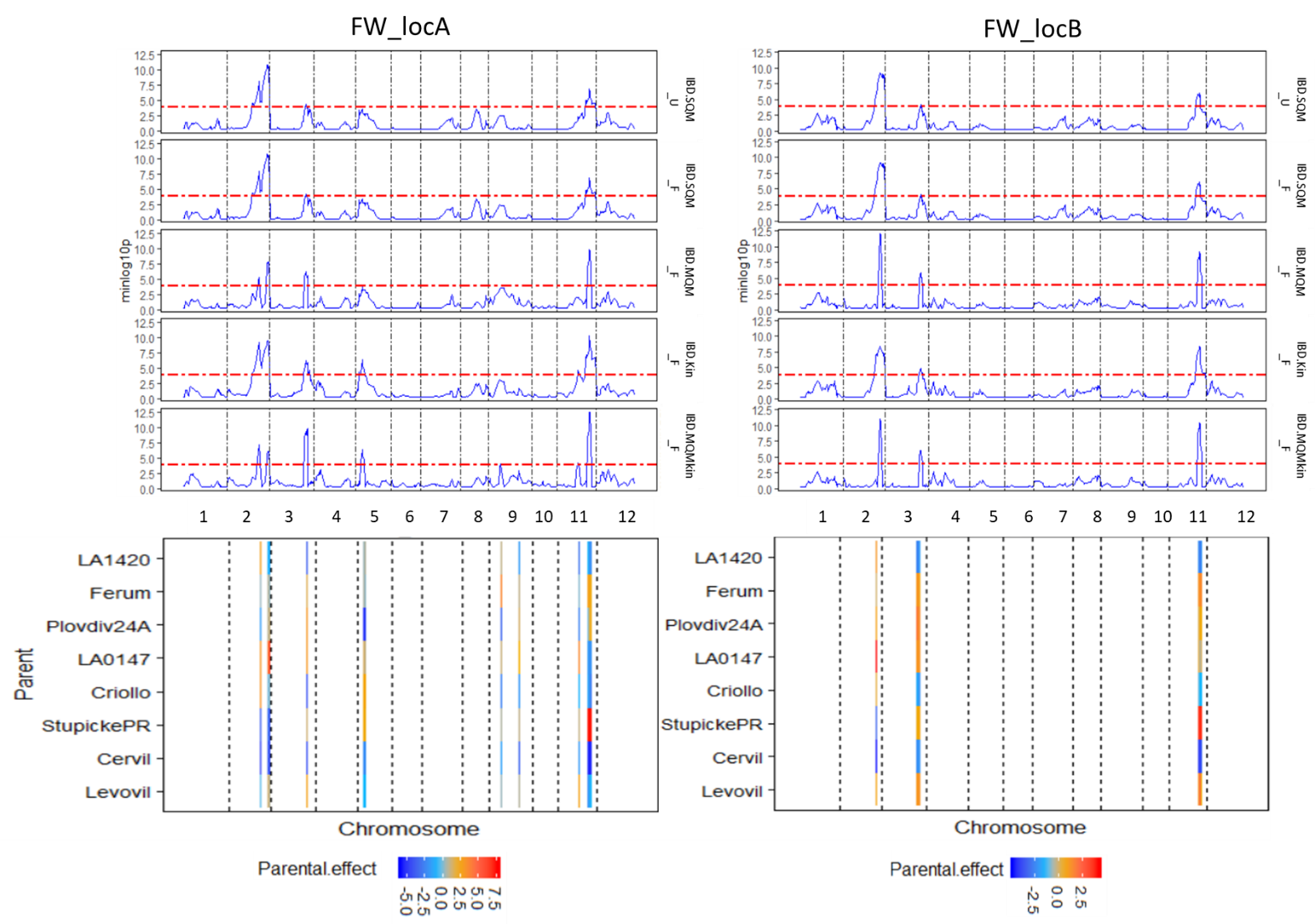

Supplement: Supplementary file 1 — Supplementary file1 (DOCX 2744 KB) [file 122_2021_3919_MOESM1_ESM.docx]
