# Supplementary material for: An IBD-based mixed model approach for QTL mapping in multiparental populations
Source: Theor Appl Genet. 2021 Aug 3;134(11):3643–60. doi: 10.1007/s00122-021-03919-7 (PMC8519866; doi:10.1007/s00122-021-03919-7)
Supplement: Supplementary file 2 — Supplementary file2 (DOCX 20 KB) [file 122_2021_3919_MOESM2_ESM.docx]

Table S1 Summary of QTL mapping results of empirical MPP designs. The number of QTLs and BIC apply to the final multi-QTL model fitted based on genome scans with mentioned IBD-based mixed models.

| Trait | IBD-based  mixed models | Number of  identified QTLs | BIC |
| --- | --- | --- | --- |
| **Maize diallel** | | | |
| GDDTAP | *IBD.SQM_U* | 3 | 4522 |
|  | *IBD.SQM_F* | 3 | 4479 |
|  | *IBD.MQM_F* | 6 | 4416 |
|  | *IBD.Kin_F* | 7 | 4404 |
|  | *IBD.MQMkin_F* | 7 | 4401 |
|  |  |  |  |
| GDDTSP | *IBD.SQM_U* | 2 | 4846 |
|  | *IBD.SQM_F* | 3 | 4803 |
|  | *IBD.MQM_F* | 6 | 4707 |
|  | *IBD.Kin_F* | 6 | 4689 |
|  | *IBD.MQMkin_F* | 6 | 4695 |
|  |  |  |  |
| GDDTASIP | *IBD.SQM_U* | 1 | 3937 |
|  | *IBD.SQM_F* | 1 | 3928 |
|  | *IBD.MQM_F* | 1 | 3928 |
|  | *IBD.Kin_F* | 2 | 3881 |
|  | *IBD.MQMkin_F* | 2 | 3881 |
|  |  |  |  |
| PHP | *IBD.SQM_U* | 2 | 3388 |
|  | *IBD.SQM_F* | 2 | 3398 |
|  | *IBD.MQM_F* | 2 | 3397 |
|  | *IBD.Kin_F* | 5 | 3356 |
|  | *IBD.MQMkin_F* | 4 | 3354 |
|  |  |  |  |
| EHP | *IBD.SQM_U* | 3 | 2835 |
|  | *IBD.SQM_F* | 3 | 2852 |
|  | *IBD.MQM_F* | 3 | 2852 |
|  | *IBD.Kin_F* | 3 | 2842 |
|  | *IBD.MQMkin_F* | 3 | 2842 |
|  |  |  |  |
| TLNP | *IBD.SQM_U* | 3 | 224 |
|  | *IBD.SQM_F* | 3 | 243 |
|  | *IBD.MQM_F* | 4 | 225 |
|  | *IBD.Kin_F* | 3 | 197 |
|  | *IBD.MQMkin_F* | 4 | 201 |
| ***Maize NAM*** | | | |
| DGY | *IBD.SQM_U* | 3 | 4791 |
|  | *IBD.SQM_F* | 2 | 4842 |
|  | *IBD.MQM_F* | 5 | 4819 |
|  | *IBD.Kin_F* | 5 | 4698 |
|  | *IBD.MQMkin_F* | 5 | 4698 |
|  |  |  |  |
| PH | *IBD.SQM_U* | 8 | 4289 |
|  | *IBD.SQM_F* | 8 | 4314 |
|  | *IBD.MQM_F* | 9 | 4312 |
|  | *IBD.Kin_F* | 10 | 4219 |
|  | *IBD.MQMkin_F* | 11 | 4225 |
| ***Maize MAGIC*** | | | |
| PS | *IBD.SQM_U* | 1 | 669 |
|  | *IBD.SQM_F* | 1 | 669 |
|  | *IBD.MQM_F* | 1 | 669 |
|  | *IBD.Kin_F* | 1 | 672 |
|  | *IBD.MQMkin_F* | 1 | 672 |
|  |  |  |  |
| PH | *IBD.SQM_U* | 1 | 663 |
|  | *IBD.SQM_F* | 1 | 663 |
|  | *IBD.MQM_F* | 1 | 663 |
|  | *IBD.Kin_F* | 1 | 667 |
|  | *IBD.MQMkin_F* | 1 | 667 |
|  |  |  |  |
| GY | *IBD.SQM_U* | 1 | 648 |
|  | *IBD.SQM_F* | 1 | 648 |
|  | *IBD.MQM_F* | 1 | 648 |
|  | *IBD.Kin_F* | 1 | 647 |
|  | *IBD.MQMkin_F* | 1 | 647 |
| ***Tomato diallel*** | | | |
| Fruit shape | *IBD.SQM_U* | 1 | 1429 |
|  | *IBD.SQM_F* | 3 | 1429 |
|  | *IBD.MQM_F* | 2 | 1433 |
|  | *IBD.Kin_F* | 3 | 1433 |
|  | *IBD.MQMkin_F* | 3 | 1431 |
| ***Tomato NAM*** | | | |
| Disease score | *IBD.SQM_U* | 2 | 145 |
|  | *IBD.SQM_F* | 2 | 165 |
|  | *IBD.MQM_F* | 2 | 165 |
|  | *IBD.Kin_F* | 2 | 165 |
|  | *IBD.MQMkin_F* | 2 | 165 |
| ***Tomato MAGIC*** | | | |
| FT_locA | *IBD.SQM_U* | 3 | 2719 |
|  | *IBD.SQM_F* | 3 | 2719 |
|  | *IBD.MQM_F* | 4 | 2703 |
|  | *IBD.Kin_F* | 5 | 2667 |
|  | *IBD.MQMkin_F* | 8 | 2649 |
|  |  |  |  |
| FT_locB | *IBD.SQM_U* | 3 | 2346 |
|  | *IBD.SQM_F* | 3 | 2346 |
|  | *IBD.MQM_F* | 3 | 2346 |
|  | *IBD.Kin_F* | 3 | 2327 |
|  | *IBD.MQMkin_F* | 3 | 2327 |
